# Smoking increased risk of ARDS in surgical critically ill patients: results from the multicenter THAI-SICU study

**DOI:** 10.1186/cc14318

**Published:** 2015-03-16

**Authors:** P Wacharasint, P Fuengfoo, N Sukcharoen, R Rangsin, THAI-SICU Trial Group

**Affiliations:** 1 Phramongkutklao Hospital, Bangkok, Thailand; 2 Phramongkutklao College of Medicine, Bangkok, Thailand; 3 THAI-SICU Collaborating Centre, Bangkok, Thailand

## Introduction

Cigarette smoking slowly and progressively damages the respiratory system [1]. In surgical critically ill patients, whether active cigarette smoking until admission to the surgical intensive care unit (SICU) is associated with increased risk of acute respiratory distress syndrome (ARDS) is not clearly identified.

## Methods

We conducted a cohort study using the THAI-Surgical Intensive Care Unit (THAI-SICU) study databases [2], which recruited 4,652 Thai patients admitted to the SICUs from nine university-based hospitals in Thailand (April 2011 to November 2012). The enrolled patients were divided into three groups (active smokers, exsmokers, and nonsmokers). Primary outcome was the incidence of patients diagnosed with ARDS and the secondary outcomes included 28-day mortality, incidence of systemic inflammatory response syndrome (SIRS), SICU length of stay (LOS), and total SICU cost.

## Results

Of those 4,652 patients, there were 2,947 nonsmokers, 1,148 exsmokers, and 557 active smokers. There was no difference of APACHE II score between three groups of patients. The active smokers exhibited the highest incidence of ARDS (active smokers 5.4%, exsmokers 4.8%, and nonsmokers 3%, *P *= 0.003). There was no difference of 28-day mortality between the three groups of patients. Active smokers had the highest incidence of SIRS (active smokers 41%, exsmokers 37%, and nonsmokers 34%, *P *= 0.006). Compared with nonsmokers and exsmokers, active smokers had a longer SICU LOS (*P *< 0.01) and higher total SICU cost (*P *= 0.02). Patients who smoked more than 15 pack-years were 2.5 times more likely to develop ARDS than patients who smoked ≤15 pack-years (95% CI: 1.65 to 3.66, *P *< 0.001). In multivariate analysis we found that every 1 pack-year of cigarette smoking before admission to the SICU is associated with increased risk of new ARDS with a hazard ratio of 1.02 (95% CI: 1.01 to 1.02, *P *= 0.001) after adjustment for APACHE II score, age, gender, and chronic obstructive pulmonary disease.

## Conclusion

In surgical critically ill patients, active smokers are associated with increased risk of new ARDS, longer SICU LOS, and higher total ICU cost, compared with exsmokers and nonsmokers. Our findings emphasize the essential need for a smoking cessation program.

## References

[B1] Hoshino Y Am J Physiol Lung Cell Mol Physiol 2001 281 L509 16 11435227 10.1152/ajplung.2001.281.2.L509

[B2] Chittawatanarat K J Med Assoc Thai 2014 97 Suppl 1 S45 54 24855842

